# COVID-19: A Retrospective Cohort Study of Nosocomial Transmission in a District General Hospital

**DOI:** 10.7759/cureus.31245

**Published:** 2022-11-08

**Authors:** Hamza Duffaydar, Amanda Beaumont, My Chi Pham, Kiran Khamb, Aiden Plant

**Affiliations:** 1 College of Medical and Dental Sciences, University of Birmingham, Birmingham, GBR; 2 Department of Trauma and Orthopaedics, Royal Wolverhampton NHS Trust, Wolverhampton, GBR; 3 Department of Infection Prevention and Control, Walsall Healthcare NHS Trust, Walsall, GBR; 4 Department of Microbiology, Walsall Manor Hospital, Walsall, GBR

**Keywords:** coronavirus disease 2019, hospital-acquired infection, hospital acquired, nosocomial, coronavirus, covid-19

## Abstract

Background and objectives

Since the outbreak of coronavirus disease 2019 (COVID-19) in the UK, there has been concern that a higher proportion of COVID-19 deaths among inpatients were a result of nosocomial infections. We sought to investigate the proportion of nosocomial COVID-19 infections within our hospital and patient outcomes within this category.

Methods

This was a retrospective cohort study of 616 patients admitted to the hospital and tested positive for SARS-CoV-2 through a polymerase chain reaction test with particular emphasis on 104 patients who were classed as probable or definite hospital-acquired COVID-19. Demographic and clinical data were extracted from the electronic records of patients, and the outcome of their stay was recorded.

Results

The median (interquartile range) age of inpatients testing positive for SARS-CoV-2 was 76 (62, 84) years, and the ethnic breakdown of patients was similar to that of the local population. Inpatient mortality was similar to other hospitals in the UK at 41%. Patients with a hospital-acquired infection were older, with a median age of 79 (69, 86) years, more likely to be of White ethnicity, and more likely to die in the hospital.

Conclusion

Older age was associated with a higher risk of healthcare-associated infection, and as a result, patients were more likely to die.

## Introduction

In December 2019, a respiratory tract illness, which we are now familiar with as the novel pathogenic strain of coronavirus, was reported as a cluster of cases in Wuhan, China [1]. The coronavirus disease 2019 (COVID-19) is caused by the severe acute respiratory syndrome coronavirus 2 (SARS-CoV-2). Since then, the World Health Organization (WHO) has declared a state of a public health emergency, and as of 6 November 2022, over 637 million cases were confirmed in 216 countries with more than 6,605,464 confirmed deaths from the disease [2,3].

The first laboratory-confirmed case of COVID-19 in the UK was reported on 30 January 2020 with a subsequent exponential rise in cases nationally; as of 11 September 2022, 993,738 patients had tested positive for the disease with 165,953 deaths [4].

The first COVID-19 case in Walsall was reported on 5 March 2020. The Metropolitan Borough of Walsall, with an estimated population of 285,500 inhabitants, is ethnically diverse and one of the most socially deprived in the country [5]. At present, there exists a concern that COVID-19 deaths are disproportionately affecting members from ethnic minority groups or a background of social deprivation [6]. This makes Walsall a highly relevant population to investigate. Walsall Manor, a busy suburban hospital with 488 beds, confirmed its first polymerase chain reaction (PCR)-positive COVID-19 inpatient on 10 March 2020. Since then, cases have risen rapidly to a total of 619 laboratory-confirmed COVID-19 inpatients by 17 June 2020. The peak number of daily deaths amongst COVID-19 inpatients was on 3 April 2020 with 12 deaths.

Nosocomial infections, also known as healthcare-associated infections (HCAIs), are infections acquired in hospitals. They can be acquired from healthcare workers or through direct or indirect contact with other patients [7]. Given the virulence and transmissibility of this virus, there was a concern that a high proportion of COVID-19 deaths amongst inpatients was a result of nosocomial infections. Hence, we sought to investigate the proportion of nosocomial infections in our hospital and the category of patients in this group. We hypothesised that amongst patients who acquired COVID-19 whilst in hospital, patients who died were more likely to be older with multiple co-morbidities.

Since the early case reports, there have been several publications on the presenting features and outcomes of the disease [8,9]. However, few publications have addressed COVID-19 as a hospital-acquired infection, particularly within the UK. Given the importance and significant burden of healthcare-acquired infections, tailored, evidence-based infection prevention measures are a priority to avoid a resurgence of nosocomial COVID-19 cases in the event of increasing community transmission.

## Materials and methods

All admitted inpatients to the hospital with a positive test for SARS-CoV-2 PCR obtained from a nasopharyngeal swab from 5 March 2020 to 7 June 2020 were included. Patients with a clinical diagnosis of COVID-19 but negative PCR tests and those treated in a community setting were excluded from the sample.

The estimated median incubation period of COVID-19 is five days, with around 1% of cases developing symptoms after 14 days [10]. Therefore, for the purpose of analysis, the patients have been divided into the following groups defined by NHS England and Improvement [11]: (i) definite community-acquired COVID-19 - patients returning a positive PCR test within the first 48 hours of their admission; (ii) probable community-acquired COVID-19 - patients returning a positive PCR test between three and seven days after their admission; (iii) probable hospital-acquired COVID-19 - patients returning a positive PCR test between eight and 14 days after their admission; (iv) definite hospital-acquired COVID-19 - patients returning a positive PCR test 15 days or more after their admission.

**Figure 1:**
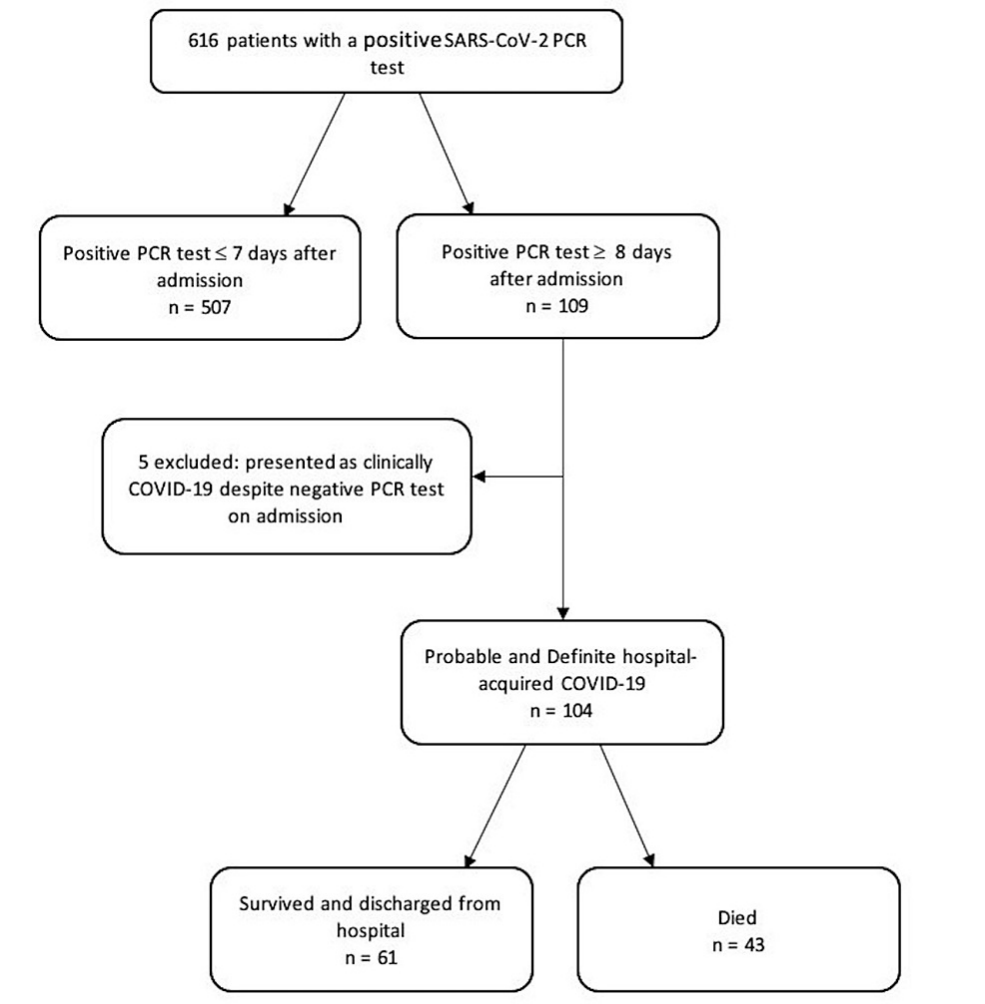
Flowchart with inclusion criteria for nosocomial transmission PCR: polymerase chain reaction.

A total of 616 inpatients had a positive SARS-CoV-2 PCR test, of which 507 tested positive less than eight days after admission. Hence, they were classified as definite or probable community-acquired COVID-19 and forgone from further analysis. A total of 109 patients had a positive PCR test obtained from samples taken eight days or more after admission. However, five patients presented clinically with COVID-19 symptoms on admission despite a negative PCR test. They returned a positive PCR test later on in their stay, but as they presented with a likely COVID-19 diagnosis on admission, they have been excluded from the cohort as they may have had a community-acquired COVID-19 infection. Our final cohort consisted of 104 inpatients classified as having either probable or definite hospital-acquired COVID-19 (Figure 1).

Data were collected retrospectively from electronic patient records. Standardised data were collected on age, gender, ethnicity, co-morbidities, past medication history, symptoms at presentation, and initial diagnosis from the current admission. The primary cause of death, as reported on the death certificate, was also recorded for deceased patients. Patients’ Vulnerability Index score was calculated using their postcode, and the British Red Cross COVID-19 Vulnerability Index was also reported. This takes into account the clinical, health and well-being, economic, and social vulnerability of the population in each neighbourhood to calculate their relative vulnerability to COVID-19 [5].

The primary outcome assessed was death vs. survival from hospital and discharge. The secondary outcome was the length of stay in the hospital before testing positive for SARS-CoV-2. Most of the variables did not follow a normal distribution and thus were reported as median (interquartile range (IQR)). Non-parametric tests were used throughout. Continuous variables were analysed using an independent t-test, and the chi-squared test was used for categorical variables. Analysis was performed by a professional statistician using Stata version 14.0 (StataCorp LLC, College Station, TX).

## Results

**Figure 2:**
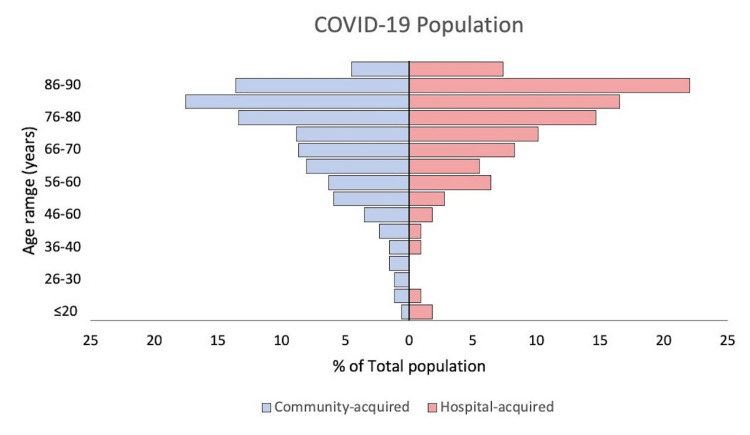
Pyramid chart with a breakdown of age for community-acquired and hospital-acquired groups as a percentage of their respective groups

As of 7 June 2020, 616 inpatients tested positive for SARS-CoV-2. The median (IQR) age of patients was 76 (62, 84) years, which is almost double of Walsall’s median age of 33 years [5]. Almost three-quarters (76%) of patients were above the age of 60 and almost one-third (37%) were above the age of 80. There was an equal number of males and females affected with a 1:1 ratio of male to female patients. The ethnicity of patients who tested positive mirrored that of the local population with 71% White, 15% Asian, 4% Black, and 1% mixed race [12]. According to the criteria set by NHS England and Improvement, 450 (73%) patients would have been classified as definite community-acquired COVID-19, 57 (9%) as probable community-acquired COVID-19, 44 (7%) as probable hospital-acquired COVID-19, and 65 (11%) as definite hospital-acquired COVID-19. Of the 616 inpatients testing positive for SARS-CoV-2, 400 (65%) survived and were discharged and 80 (35%) died. Patients who died were significantly older compared to those who survived (median (IQR) age: 80 (69, 85) vs. 72 (56.75, 83), p = 0.001). Gender ratio, ethnicity, and length of stay were not significantly different between the two groups (Figure 2).

A total of 104 patients were defined as having a probable or definite hospital-acquired COVID-19, with a length of stay of eight days or more in the hospital before testing positive for SARS-CoV-2. Table 1 summarises the demographic and clinical characteristics of these patients, subdivided by outcomes. A total of 61 (59%) patients survived and 43 (41%) patients died. Patients who died were significantly older (median (IQR) age: 82 (76, 86.5) vs. 75 (62, 86), p = 0.006). There was no association between gender and the outcome of hospital stay. There was a trend towards a difference in ethnicity, with a higher proportion of White patients amongst those who died compared to other known ethnicities (chi^2^ p = 0.003). However, this reflects a higher proportion of the local elderly population being White [12].

**Table 1:** Demographics and baseline characteristics by the outcome of hospital stay for all patients testing positive

	All patients (n = 104)	By the outcome of hospital stay		P-value
		Discharged (n = 61)	Died (n = 43)	
Demographics				
Age in years, median (interquartile range)	79 (69,86)	75 (62,86)	82 (76,86.5)	<0.01
Age breakdown, n (%)				
≤50	6 (6)	6 (10)	0 (0)	-
51-60	10 (10)	8 (13)	2 (5)	-
61-70	14 (13)	10 (16)	4 (9)	-
71-80	27 (26)	13 (21)	14 (33)	-
81-90	39 (38)	20 (33)	19 (44)	-
>90	8 (8)	4 (7)	4 (9)	-
Male gender, n (%)	52 (50)	32 (52)	17 (40)	0.550
Ethnicity, n (%)				
Asian	4 (4)	4 (7)	0 (0)	0.065
Black	3 (3)	3 (5)	0 (0)	
White	81 (78)	44 (72)	37 (86)	
Not available	16 (15)	10 (16)	6 (14)	
Pre-existing co-morbidities, n (%)				
Asthma	9 (9)	4 (7)	5 (12)	0.365
Chronic obstructive pulmonary disease	12 (12)	7 (11)	5 (12)	0.981
Cardiovascular	55 (53)	30 (49)	25 (58)	0.408
Diabetes	30 (29)	16 (26)	14 (33)	0.297
Hypertension	61 (59)	38 (62)	23 (53)	0.517
Renal	22 (21)	9 (15)	13 (30)	0.686
Cancer/malignancy	16 (15)	9 (15)	7 (16)	0.896
Rheumatoid	14 (13)	9 (15)	5 (12)	0.580
Endocrine	13 (13)	11 (18)	2 (5)	0.164
Cognitive	26 (25)	16 (26)	10 (23)	0.478
Other	40 (38)	22 (36)	18 (42)	0.382
Disease characteristics				
Symptoms at presentation, n (%)				
Cough	8 (8)	6 (10)	2 (5)	0.328
Shortness of breath	13 (13)	8 (13)	5 (12)	0.832
Fever	9 (9)	5 (8)	4 (9)	0.818
Abdominal symptoms	23 (22)	15 (25)	8 (19)	0.469
Generally unwell	14 (13)	7 (11)	7 (16)	0.707
Fall	39 (38)	22 (36)	17 (40)	0.719
Other	26 (25)	15 (25)	11 (26)	0.807
Initial diagnosis, n (%)				
Respiratory (not COVID-19)	17 (16)	12 (20)	5 (12)	0.275
Cardiovascular	9 (9)	2 (3)	7 (16)	0.020
Abdominal	21 (20)	16 (26)	5 (12)	0.068
Brain injury	8 (8)	4 (7)	4 (9)	0.605
Dermal pathology	7 (7)	4 (7)	3 (7)	0.933
Fracture	14 (13)	8 (13)	6 (14)	0.902
Genitourinary	16 (15)	10 (16)	6 (14)	0.734
Sepsis	3 (3)	0 (0)	3 (7)	0.036
Other	9 (9)	5 (8)	4 (9)	0.843
Medication history, n (%)				
Warfarin (and other blood thinners)	34 (33)	18 (30)	16 (37)	0.410
Antihypertensives	56 (54)	32 (52)	24 (56)	0.566
Cardiovascular medication	15 (14)	7 (11)	8 (19)	0.480
Diabetic medication	19 (18)	9 (15)	10 (23)	0.269
Diuretics	15 (14)	9 (15)	6 (14)	0.909
Statin	40 (38)	21 (34)	19 (44)	0.314
Steroids	7 (7)	6 (10)	1 (2)	0.132
Vulnerability Index score				
5	80 (77)	48 (79)	32 (74)	0.611
4	10 (10)	4 (7)	6 (14)	0.208
3	10 (10)	5 (8)	5 (12)	0.559
2	0 (0)	0 (0)	0 (0)	-
1	4 (4)	4 (7)	0 (0)	0.087

Hypertension and cardiovascular conditions were the most prevalent co-morbidities present in 61 (59%) and 55 (53%) patients, respectively. The most common presenting symptom was a fall, which 39 (38%) patients presented with, and 56 (54%) patients were on antihypertensive medications. However, there was no significant difference in pre-existing co-morbidities, symptoms at presentation, and medication history amongst patients who died and those who survived.

The only significant difference in initial diagnosis on presentation amongst the two groups was for cardiovascular conditions and sepsis. Patients who died were more likely to have been diagnosed as having a cardiovascular condition (7 (16%) vs. 2 (3%), chi^2^ p = 0.02) or sepsis (3 (7%) vs. 0 (0%), chi^2^ p = 0.036) on the concurrent hospital admission as opposed to those who survived.

Table 2 displays the demographic and clinical characteristics of the patients, subdivided into probable and definite hospital-acquired COVID-19 for patients who survived and those who died. Patients in the definite HCAI group were more likely to have died than survived (chi^2^ p = 0.041). The odds of patients from the definite HCAI group dying is 5.9 times that of survival (OR = 5.9, 95% CI: 1.86, 18.67).

**Table 2:** Demographics and baseline characteristics by the outcome of hospital admission for HCAI patients COPD: chronic obstructive pulmonary disease; HCAI: healthcare-associated infection; LoS: length of stay.

	All patients (n = 104)	By the outcome of hospital stay						P-values
		Discharged			Died			
		LoS 8-14 days (n = 23)	LoS ≥ 15 days (n = 38)	Total discharged (n = 61)	LoS 8-14 days (n = 4)	LoS ≥ 15 days (n = 39)	Total (n = 43)	
Demographics								
Age in years, median (interquartile range)	79 (69, 86)	80 (64, 86)	73.5 (62.5, 84.5)	75 (62, 86)	87.5 (81, 88.25)	82 (76, 86)	82 (76, 86.5)	0.471
Age breakdown, n (%)								
≤50	6 (6)	2 (9)	4 (11)	6 (10)	0 (0)	0 (0)	0 (0)	-
51-60	10 (10)	4 (17)	4 (11)	8 (13)	0 (0)	2 (5)	2 (5)	-
61-70	14 (13)	1 (4)	9 (24)	10 (16)	1 (25)	3 (8)	4 (9)	-
71-80	27 (26)	5 (22)	8 (21)	13 (21)	0 (0)	14 (36)	14 (33)	-
81-90	39 (38)	11 (48)	9 (24)	20 (33)	3 (75)	16 (41)	19 (44)	-
>90	8 (8)	0 (0)	4 (11)	4 (7)	0 (0)	4 (10)	4 (9)	-
Male gender, n (%)	52 (50)	12 (52)	20 (53)	32 (52)	4 (100)	13 (33)	17 (40)	0.418
Ethnicity, n (%)								
Asian	4 (4)	3 (13)	1 (3)	4 (7)	0 (0)	0 (0)	0 (0)	<0.01
Black	3 (3)	2 (9)	1 (3)	3 (5)	0 (0)	0 (0)	0 (0)	
White	81 (78)	16 (70)	28 (74)	44 (72)	4 (100)	33 (85)	37 (86)	
Not available	16 (15)	2 (9)	8 (21)	10 (16)	0 (0)	6 (15)	6 (14)	
Pre-existing comorbidities, n (%)								
Asthma	9 (9)	2 (9)	2 (5)	4 (7)	0 (0)	5 (13)	5 (12)	0.820
COPD	12 (12)	2 (9)	5 (13)	7 (11)	0 (0)	5 (13)	5 (12)	0.570
Cardiovascular	55 (53)	13 (57)	17 (45)	30 (49)	1 (25)	24 (62)	25 (58)	0.252
Diabetes	30 (29)	9 (39)	7 (18)	16 (26)	1 (25)	13 (33)	14 (33)	0.330
Hypertension	61 (59)	17 (74)	21 (55)	38 (62)	4 (100)	19 (49)	23 (53)	0.014
Renal	22 (21)	1 (4)	8 (21)	9 (15)	2 (50)	11 (28)	13 (30)	0.064
Gastro	31 (30)	3 (13)	15 (39)	18 (30)	3 (75)	10 (26)	13 (30)	0.276
Cancer/malignancy	16 (15)	1 (4)	8 (21)	9 (15)	1 (25)	6 (15)	7 (16)	0.276
Rheumatoid	14 (13)	6 (26)	3 (8)	9 (15)	0 (0)	5 (13)	5 (12)	0.260
Endocrine	13 (13)	4 (17)	7 (18)	11 (18)	0 (0)	2 (5)	2 (5)	0.753
Cognitive	26 (25)	8 (35)	8 (21)	16 (26)	0 (0)	10 (26)	10 (23)	0.515
Other	40 (38)	7 (30)	15 (39)	22 (36)	2 (50)	16 (41)	18 (42)	0.791
Disease characteristics								
Symptoms at presentation, n (%)								
Cough	8 (8)	4 (17)	2 (5)	6 (10)	0 (0)	2 (5)	2 (5)	0.256
Shortness of breath	13 (13)	4 (17)	4 (11)	8 (13)	0 (0)	5 (13)	5 (12)	0.564
Fever	9 (9)	2 (9)	3 (8)	5 (8)	0 (0)	4 (10)	4 (9)	0.873
Abdominal symptoms	23 (22)	6 (26)	9 (24)	15 (25)	1 (25)	7 (18)	8 (19)	0.580
Generally unwell	14 (13)	3 (13)	4 (11)	7 (11)	1 (25)	6 (15)	7 (16)	0.536
Fall	39 (38)	8 (35)	14 (37)	22 (36)	2 (50)	15 (38)	17 (40)	0.388
Other	26 (25)	5 (22)	10 (26)	15 (25)	1 (25)	10 (26)	11 (26)	0.160
Initial diagnosis, n (%)								
Respiratory not COVID-19	17 (16)	6 (26)	6 (16)	12 (20)	0 (0)	5 (13)	5 (12)	0.417
Cardiovascular	9 (9)	1 (4)	1 (3)	2 (3)	1 (25)	6 (15)	7 (16)	0.820
Abdominal	21 (20)	5 (22)	11 (29)	16 (26)	1 (25)	4 (10)	5 (12)	0.661
Brain injury	8 (8)	0 (0)	4 (11)	4 (7)	0 (0)	4 (10)	4 (9)	0.173
Dermal pathology	7 (7)	2 (9)	2 (5)	4 (7)	0 (0)	3 (8)	3 (7)	0.889
Fracture	14 (13)	2 (9)	6 (16)	8 (13)	0 (0)	6 (15)	6 (14)	0.407
Genitourinary	16 (15)	5 (22)	5 (13)	10 (16)	1 (25)	5 (13)	6 (14)	0.316
Sepsis	3 (3)	0 (0)	0 (0)	0 (0)	1 (25)	2 (5)	3 (7)	0.922
Other	9 (9)	2 (9)	3 (8)	5 (8)	0 (0)	4 (10)	4 (9)	0.302
Medication history, n (%)								
Warfarin (and other blood thinners)	34 (33)	10 (43)	8 (21)	18 (30)	1 (25)	15 (38)	16 (37)	0.580
Antihypertensives	56 (54)	15 (65)	17 (45)	32 (52)	3 (75)	21 (53)	24 (56)	0.302
Cardiovascular medication	15 (14)	1 (4)	6 (16)	7 (11)	1 (25)	7 (18)	8 (19)	0.369
Diabetic medication	19 (18)	6 (26)	3 (8)	9 (15)	0 (0)	10 (26)	10 (23)	0.613
Diuretics	15 (14)	1 (4)	8 (21)	9 (15)	1 (25)	5 (13)	6 (14)	0.337
Statin	40 (38)	9 (39)	12 (32)	21 (34)	2 (50)	17 (44)	19 (44)	0.949
Steroids	7 (7)	2 (9)	4 (11)	6 (10)	0 (0)	1 (3)	1 (2)	0.165
Cause of death								
COVID-19 pneumonia	n/a	n/a	n/a	n/a	2 (50)	25 (64)	27 (63)	0.651
Other pneumonia	n/a	n/a	n/a	n/a	0 (0)	4 (10)	4 (9)	0.905
Other respiratory	n/a	n/a	n/a	n/a	0 (0)	1 (3)	1 (2)	0.905
Cardiac arrest	n/a	n/a	n/a	n/a	0 (0)	1 (3)	1 (2)	0.905
Renal cause	n/a	n/a	n/a	n/a	0 (0)	1 (3)	1 (2)	0.905
Other	n/a	n/a	n/a	n/a	0 (0)	3 (8)	3 (7)	0.163
Unknown	n/a	n/a	n/a	n/a	2 (50)	4 (10)	6 (14)	0.080
Vulnerability Index score								
5	80 (77)	21 (91)	27 (71)	48 (79)	4 (100)	28 (72)	32 (74)	0.039
4	10 (10)	0 (0)	4 (11)	4 (7)	0 (0)	6 (15)	6 (14)	0.106
3	10 (10)	2 (9)	3 (8)	5 (8)	0 (0)	5 (13)	5 (12)	0.744
2	0 (0)	0 (0)	0 (0)	0 (0)	0 (0)	0 (0)	0 (0)	-
1	4 (4)	0 (0)	4 (11)	4 (7)	0 (0)	0 (0)	0 (0)	0.430

There is no significant difference in age and gender amongst patients with a probable or definite HCAI in both survived and died groups, although those patients who died were more likely to be older. A higher proportion of White patients were found to be in both groups of people who died compared to those who survived, and this was statistically significant (p = 0.001). Sixteen (70%) patients in the probable HCAI and 28 (74%) patients in the definite HCAI group who survived were White compared to four (100%) and 33 (85%) patients in those who died.

There was also a statistically significant difference (p = 0.014) amongst patients having hypertension as a pre-existing co-morbidity. The results show that those in both the survived and died probable HCAI group were more likely to have hypertension compared to those in the survived and died definite HCAI group. All other pre-existing co-morbidities, symptoms at presentation, initial diagnoses, medication history, and cause of death were not significantly different amongst the four groups. However, the most common primary cause of death as listed on the death certificate of those who died remains COVID-19 pneumonia, listed on the death certificate of 27 (63%) patients.

There was a significant difference in patients residing in areas listed as having the highest vulnerability score as per the British Red Cross COVID-19 Vulnerability Index. A total of 21 (91%) and 27 (71%) patients in the probable and definite HCAI survived group were from areas with the highest Vulnerability Index score as compared to four (100%) and 28 (72%) in the probable and definite HCAI died category (p = 0.039). Furthermore, 25 (93%) patients in the probable HCAI group were from areas with the highest vulnerability score for social deprivation compared to 55 (71%) of patients in the definite HCAI group (chi^2^ p = 0.025). Whilst there was no significant difference across the COVID-19 Vulnerability Index scores amongst patients who died and those who survived, in both categories, three-quarters of patients came from areas with the highest vulnerability score. This reflects the high prevalence of social deprivation within the area of Walsall [5].

## Discussion

We reported here the demographics of 616 patients with confirmed COVID-19 presenting to our hospital with a focus on 104 patients that were deemed to have acquired COVID-19 whilst in the hospital up to 7 June 2020.

In-hospital mortality per admission was high at 35%, in line with the International Severe Acute Respiratory and Emerging Infection Consortium (ISARIC) collaboration, which reports a mortality rate of 33% across 166 hospitals in the UK [13]. However, the mortality rate amongst COVID-19 inpatients differs significantly from reports from China, with a reported mortality rate of 1.4% [14], and that of the USA at 21%, as reported by Richardson et al. [9]. However, the recommended practice in the UK has been to only admit patients requiring hospitalisation as they are more unwell. In China, especially at the start of the pandemic, the current practice was to admit all patients who tested positive for SARS-CoV-2 regardless of the severity of their condition. The UK is also known for having an ageing population with a median age of 40.5 years as compared to the USA and China, both with a median age of 38.4 years [15]. Age has been shown to be independently linked to mortality amongst COVID-19-positive patients [13].

Of inpatients testing positive for SARS-CoV-2, 17% were classified as having a hospital-acquired infection according to the NHS England and Improvement criteria [11]. This is similar to those reported in studies carried out across hospitals in the East of England NHS Trust (14%) [16] and University College London Hospital NHS Trust (15%) [17]. The rates of nosocomial infections in UK hospitals are significantly lower compared to those reported in a meta-analysis by Zhou et al. conducted in the Hubei province [18]. They reported 44% of COVID-19 cases to be of hospital origin. This could potentially reflect the robust infection control measures in UK hospitals to limit the spread of nosocomial infections and the fact that the UK had relatively more time to prepare for an outbreak compared to China. However, it must be noted that the meta-analysis by Zhou et al. only included hospitals in the Hubei province and the authors admitted that their results were based on “low-quality research”, and thus the credibility of their results is low. It is also interesting to note that as of 17 July 2020, 24% of Walsall hospital staff had positive antibodies for SARS-CoV-2, which is quite similar to the rate of nosocomial infection for COVID-19 within the hospital.

There was a disproportionate number of elderly patients who tested positive for COVID-19 within the hospital. Three-quarters of patients testing positive up to 7 June 2020 were above 60 years and one-third were above the age of 80 years. This is significantly different to the local population where 23% and 4% of local residents were above the ages of 60 and 80, respectively, according to the 2011 population census [12]. This reflects the evidence that the elderly are more susceptible to the virus, have more severe symptoms, and thus, if infected, are more likely to be hospitalised [14]. There was a further disparity in age among those who were classified as having a nosocomial infection. In the HCAI group, 85% of patients were over 60 and 46% were over 80 years old. This could potentially also show that the elderly are at a higher risk of acquiring COVID-19 within the hospital as compared to the general population.

Whilst there is much concern in the UK that Black, Asian, and minority ethnic (BAME) groups are disproportionately affected and more likely to die from COVID-19, our data do not reflect this. Of patients testing positive for SARS-CoV-2 with known ethnicity, 78% were White, 17% were Asian, and 4% were Black. This is almost identical to the wider Walsall Borough where 79% are White, 15% are Asian, and 2% are Black, according to the 2011 population census [19]. Amongst the patients with a nosocomial infection, however, 92% were White, 5% were Asian, and 3% were Black if their ethnicity was known. This shows that people of White ethnicity were more likely to have acquired COVID-19 in the hospital whilst the converse applies to those of Asian ethnicity. However, the population of those with an HCAI was older than the general population. The 2011 population census shows that a higher proportion of the elderly population in Walsall is White compared to the total population and ethnic minorities are more likely to be in the younger age groups [12].

Furthermore, there is also increasing evidence that people from lower socio-economic backgrounds are at higher risk of contracting COVID-19 and dying [19]. More than three-quarters of patients in the HCAI group are from an area listed as having the highest vulnerability index score as per the British Red Cross COVID-19 Vulnerability Index. Whilst there are no data to compare with, this perhaps shows that on the whole, patients living in more deprived areas are at a higher risk of acquiring COVID-19 both within the community and the hospital. It must also be noted that the Walsall Borough is known for having several socially deprived areas, and thus the results could simply be representative of the local population [6].

There are several limitations to this study. We did not collect data on all the patients who tested positive for SARS-CoV-2 but instead concentrated our efforts on those in the HCAI group. As this was an observational study, data collection was not standardised. There were also some data missing on the electronic records of patients, and whilst few, this could have influenced the results obtained. However, despite these limitations, within the circumstances, our cohort has allowed us to gain an accurate depiction of nosocomial COVID-19 within our hospital.

## Conclusions

We have described the patients who have acquired COVID-19 whilst in our suburban hospital located in Walsall. The rate of nosocomial infection is consistent with that reported across other hospitals in the UK. Patients who were classed as definite HCAI were significantly more likely to die. There was also a higher proportion of elderly and White patients who were within the HCAI group.
